# Association Between Non-high-density Lipoprotein Cholesterol and 3-Month Prognosis in Patients With Spontaneous Intracerebral Hemorrhage

**DOI:** 10.3389/fneur.2020.00920

**Published:** 2020-08-21

**Authors:** Hao Feng, Xin Wang, Wenjuan Wang, Xingquan Zhao

**Affiliations:** ^1^Department of Neurology, Beijing Tiantan Hospital, Capital Medical University, Beijing, China; ^2^China National Clinical Research Center for Neurological Diseases, Beijing, China; ^3^Center of Stroke, Beijing Institute for Brain Disorders, Beijing, China; ^4^Beijing Key Laboratory of Translational Medicine for Cerebrovascular Disease, Beijing, China

**Keywords:** spontaneous, intracerebral hemorrhage, non-high-density lipoprotein cholesterol, 3-month, prognosis

## Abstract

**Background:** Previous studies have indicated a significant correlation between cholesterol levels and the incidence and outcomes of intracerebral hemorrhage (ICH), However, the association between non-high-density lipoprotein cholesterol (non-HDLC) levels and ICH functional outcomes are still unclear.

**Method:** We included 654 consecutive spontaneous ICH patients who were enrolled in a prospective registry. We collected clinical, demographic, and laboratory data using standardized forms, and non-HDLC levels and 3-month modified Rankin Scale (mRS) scores were recorded. We performed multivariate logistic regression and interaction analyses to explored the association between non-HDLC levels and ICH functional outcomes.

**Results:** Of 654 patients included in the study, 281 (42.9%) had poor functional outcome. Univariate analysis showed that high non-HDLC level was associated with good functional outcome at 90 days (*p* = 0.001). After adjustment for confounding factors, a high non-HDLC level (≥154.89 mg/dl) remained as an indicator of good functional outcome at 90 days [multivariate-adjusted odds ratios (OR) 0.50, 95%CI 0.27–0.92; p-value for trend = 0.043], and was stronger for female patients (OR: 0.13, 95%CI: 0.03–0.50).

**Conclusion:** ICH patients with higher non-HDLC levels had a decreased prevalence of poor functional outcome at 90 days, and a high non-HDLC level is an independent indicator of good functional outcome at 90 days from onset, especially in females.

## Introduction

Intracerebral hemorrhage (ICH) is a devastating neurological disease that accounts for 10–15% of all stroke types (1, 2). ICH has a high mortality rate and a poor prognosis, with a median mortality rate of 40.4% at 1 month (1). Only 12 - 39% of survivors could achieve long-term functional independence (3).

Many studies regarding the effect of serum lipids on both ischemic and hemorrhagic stroke have been published (4, 5). Li et al. noted that low total cholesterol (TC) levels are strong independent predictors of poor outcomes at 3 months in both acute ischemic stroke and ICH patients (6). Vauthey et al. (7) found that high TC levels were associated with a better functional outcome in the first month after ischemic stroke, however, Weir et al. (8) observed that serum TC levels were not an independent predictor of stroke outcome.

Atherosclerosis and lipoprotein metabolism disorders are important pathological changes leading to ischemic stroke. Non-high-density lipoproteins (non-HDLC) include very-low-density lipoprotein (VLDL), low density lipoprotein (LDL), medium density lipoprotein (IDL), and lipoprotein (a) (9). Recent studies showed that non-HDLC seems to have a better predictive value for the occurrence of coronary heart disease (CHD) than LDL (10–13).

A recent cohort study from China found that non-HDLC levels have predictive values for ischemic stroke (14). However, the association between non-HDLC and 3-month functional outcome of ICH is still unclear. Therefore, we aimed to investigate the association between serum non-HDLC levels and 3-month functional outcomes after ICH in this prospective cohort study.

## Methods

The study protocol was approved by the Institutional Review Board (IRB) of the Beijing Tiantan Hospital affiliated to Capital Medical University. The ethics committee(s) approved consent by proxy in the ethics statement.

### Inclusion and Exclusion Criteria

The inclusion criteria were: (1) the first CT scan was done within 24 h from the time of symptom onset, (2) non-HDLC levels were recorded in the database, and (3) patients older than 18 years old. The exclusion criteria were: (1) primary ventricular hemorrhage, (2) secondary ICH caused by trauma, tumor, aneurysm, cavernous hemangioma, arteriovenous malformations, acute thrombolysis, or coagulopathy, or (3) surgical intervention (including extraventricular drainage, craniotomy, hematoma puncture and aspiration, etc.) during the follow-up. We also excluded patients with anticoagulant therapy before symptom onset.

### Baseline Information

For each patient, trained stroke physicians collected detailed patient information, clinical evaluation, and history of stroke risk factors using standard questionnaires at baseline. Arterial hypertension was diagnosed when its presence was documented in medical records or when at least two blood pressure readings were higher than 140 mmHg (systolic) or higher than 90 mmHg (diastolic) after the acute phase of stroke. Diabetes was recorded as present when a patient had known diabetes mellitus on admission, or when the plasma glucose value was higher than 11 mmol/l on admission or during the hospital stay. Smoking was documented when a patient smoked any kind of tobacco daily, and former and current smokers were both classified as smokers. Alcohol intake was noted when consumed daily.

We analyzed variables including age, sex, past medical history, National Institutes of Health Stroke Scale (NIHSS) on admission, Glasgow Coma Scale (GCS) on admission, blood pressure on admission, ICH score, and secondary intraventricular hemorrhage. On the initial CT scan, we evaluated ICH localization, volume (ABC/2 method) and infratentorial affectation. Routine laboratory testing was performed with in 1 h of arrival. Blood samples were collected from an antecubital vein the next morning following an overnight fasting (>8 h) and levels of serum TC, HDL, and LDL in serum were recorded. Non-HDLC levels were calculated by subtracting serum HDL levels from serum TC levels. We also documented prior antiplatelet/statin use and statin use after admission. In this study, statin use was determined by the stroke physicians according to the patient's blood lipid status and general clinical condition and we do not have a standard prescribing routine for statin use after admission.

### Follow-Up Information and ICH Clinical Outcomes

At 90 days after ICH onset, the patients' functional outcomes were assessed based on modified Rankin Scale (mRS) by telephone interviews. Follow-up telephone interviews were utilized for all included patients. The interviewers were blinded to prognostic factors during the follow-up evaluation and were trained on the interview protocol. For patients who were not reached at the first telephone follow-up interview, we conducted follow-up telephone interviews once a week for 3 weeks. If none of the four telephone follow-up interviews were successful, the follow-up was considered as lost and the mRS at discharge would be taken as the mRS at 90 days from onset. Patients with mRS scores ≥ 3 were classified to the poor functional outcome group and those with mRS scores <3 were classified to the good functional outcome group.

### Statistical Analyses

Statistics were performed with the SPSS software (version 24.0; IBM, Armonk, NY). We used median (interquartile range) to describe continuous variables and percentage for categorical variables. We used the Student's *t*-test for non-paired samples for the comparison of normally distributed parameters and the Wilcoxon test for the comparison of non-parametric variables. The Chi-squared test was applied for the comparison of categorical variables. Multivariate logistic regression analyses were used to calculate odds ratios (OR) and 95% confidence intervals (CI) for the associations of non-HDLC levels with poor functional outcomes adjusted for age, sex, history of ICH, NIHSS score on admission, GCS score on admission, glucose on admission, white blood cells (WBC) on admission, platelets on admission, hematoma volume, statins use after admission, and ICH score. A trend test was performed after the non-HDLC level was entered into the model and treated as a continuous variable. Additionally, sex and age were assessed for possible interactions between these variables and the relationship between non-HDLC levels and 3-month functional outcomes. In accounting for interactions between age and non-HDLC levels, we also used multivariate logistic regression analysis, we input the variables of age (grouped by 60 years), non-HDLC, and age (grouped by 60 years) ^*^ non-HDLC into the equation. If the *p*-value of age (grouped by 60 years) ^*^ non-HDLC was less than 0.05, it was considered as an interaction effect. The same approach was done for assessing the interaction between sex and serum non-HDLC levels.

All tests of significance were 2 tailed. A *p*-value < 0.05 was considered to be statistically significant.

## Results

The participants in this study were derived from a multicenter, prospective, and observational cohort study (Registration study on medical quality evaluation of cerebral hemorrhage based on etiology in Beijing area). A total of 13 hospitals in Beijing participated in the study. The study enrolled patients with ICH over 18 years of age confirmed by computer tomography (CT) scans from December 2014 to September 2016. A total of 1997 eligible ICH patients were screened, but 33 refused to participate in the study, and we eventually included 1964 ICH patients in the database.

Our final cohort included 654 patients with spontaneous ICH from 13 sites. The mean age was 57 years old, ranging from 50 to 67 years old. Males accounted for 68.5%. In terms of demographics, 34.7% were current or previous smokers, 37.5% had the alcohol-drinking habit, 72.0% had been diagnosed as hypertension, 15.3% had diabetes and 9.9% had hypercholesterolemia. In terms of medical history, 14.2% patients had cerebral infarction (CI) and 2.9% had ICH. Before symptom onset, 14.7% of subjects used a single antiplatelet agent (either Aspirin or Clopidogrel) and none of the subjects used dual antiplatelet agents. There were 40 (6.1%) patients had a prior statin use (no information about intensive statin treatment) and 69 (10.6%) patients continued or started to use statins after the ICH symptom (Table 1).

**Table 1:** Baseline characteristics of the study population according to 90-day clinical outcome.

	**Total**	**mRS < 3**	**mRS ≥ 3**	***p*-value**
Number	654	373	281	/
Age (years, median, IQR)	57 (50–67)	55 (48–64)	62 (53–72)	<0.001
Gender (female, %)	206 (31.5)	110 (29.5)	96 (34.2)	0.203
Onset to admission time (hour, median, IQR)	2.93 (1.50–6.18)	3.00 (1.50–6.89)	2.68 (1.50–5.50)	0.104
Hypertension no. (%)	471 (72.0)	260 (69.7)	211 (75.1)	0.129
Diabetes mellitus no. (%)	100 (15.3)	56 (15.0)	44 (15.7)	0.821
Hyperlipidemia no. (%)	65 (9.9)	36 (9.7)	29 (10.3)	0.777
History of CI no. (%)	93 (14.2)	48 (12.9)	45 (16.0)	0.254
History of ICH no. (%)	19 (2.9)	5 (1.3)	14 (5.0)	0.006
Smoking no. (%)	227 (34.7)	127 (34.0)	100 (35.6)	0.682
Alcohol no. (%)	245 (37.5)	150 (40.2)	95 (33.8)	0.094
Prior antiplatelet use no. (%)	96 (14.7)	48 (12.9)	48 (17.1)	0.132
Prior statin use no. (%)	40 (6.1)	25 (6.7)	15 (5.3)	0.471
NIHSS score on admission (median, IQR)	9 (3–14)	5 (2–9)	14 (10–21)	<0.001
GCS score on admission (median, IQR)	14 (12–15)	15 (14–15)	13 (9–15)	<0.001
SBP on admission (mmHg, median, IQR)	163 (150–183)	162 (150–181)	165 (150–185)	0.222
DBP on admission (mmHg, median, IQR)	96 (85–107)	97 (84–107)	95 (85–107)	0.741
Glucose on admission (median, IQR)	6.80 (5.82–8.32)	6.53 (5.61–8.16)	7.00 (6.10–8.56)	0.001
WBC on admission [1,000, (median, IQR)]	8.39 (6.54–10.89)	7.90 (6.46–10;19)	9.14 (6.70–11.77)	<0.001
Platelets on admission [1,000, (mean ± SD)]	213 ± 57	217 ± 54	208 ± 61	0.041
Creatinine on admission (mmol/l median, IQR)	64.0 (52.5–75.8)	63.9 (53.1–75.6)	64.2 (52.0–76.0)	0.847
In hospital non-HDLC (mg/dl, median, IQR)	125.39 (101.78–154.90)	130.81 (106.43–159.44)	121.52 (97.52–148.61)	0.003
Q1 (<101.78 mg/dl)	162	78 (48.1)	84 (51.9)	/
Q2 (101.78–125.39 mg/dl)	166	90 (54.2)	76 (45.8)	/
Q3 (125.39–154.89 mg/dl)	163	98 (60.1)	65 (39.9)	/
Q4 (>154.89 mg/dl)	163	107 (65.6)	56 (34.4)	/
In hospital HDLC (mg/dl, median, IQR)	47.99 (39.47–57.66)	46.05 (38.31–55.92)	50.31 (40.25–60.95)	0.002
Statins use after admission no. (%)	69 (10.6)	49 (13.1)	20 (7.1)	0.013
Hematoma volume (ml, median, IQR)	10.35 (5.00–22.00)	8.20 (3.87–15.12)	15.36 (8.00–28.61)	<0.001
Location				0.134
Supratentorial no. (%)	582 (89.0)	326 (56.0)	256 (44.0)	/
Infratentorial no. (%)	72 (11.0)	47 (65.3)	25 (34.7)	/
sIVH no. (%)	183 (28.0)	72 (19.3)	111 (39.5)	<0.001
ICH score (median, IQR)	1 (1–2)	1 (1–2)	2 (1–3)	<0.001

*CI, cerebral infarction; DBP, diastolic blood pressure; GCS, Glasgow Coma Scale; HDLC, high-density lipoprotein cholesterol; ICH, intracerebral hemorrhage; IQR, interquartile range; mRS, modified Rankin Scale; NIHSS, National Institutes of Health Stroke Scale; non-HDLC, non-high-density lipoprotein cholesterol; SBP, systolic blood pressure; sIVH, secondary intraventricular hemorrhage, WBC, white blood cells*.

### Baseline Characteristics

A total of 654 participants were enrolled in the study. Compared with those with good outcomes (373 patients), the group of subjects with poor outcomes (281 patients) had (1) more older adults (*p* < 0.001); (2) a greater proportion of history of ICH(*p* = 0.006); (3) higher NIHSS scores (*p* < 0.001); (4) higher GCS scores (*p* < 0.001); (5) higher admission glucose levels on admission (*p* = 0.001); (6) higher admission WBC levels on admission (*p* < 0.001); (7) higher platelets levels on admission (*p* = 0.041); (8) higher HDL levels (*p* = 0.003); (9) higher hematoma volumes (*p* < 0.001); (10) lower non-HDLC levels (*p* = 0.002); (11) a higher proportion of secondary intraventricular hemorrhage (*p* < 0.001); (12) higher ICH scores (*p* < 0.001); and (13) a lower proportion of statin use after admission (*p* = 0.013) (Table 1).

There were no significant differences in sex (good 110 vs. poor 96, *p* = 0.203), time from symptom to admission (good 3.00 vs. poor 2.68, *p* = 0.104), prior antiplatelet use [good 48(12.9%) vs. poor 48(17.1%) prior, *p* = 0.132] prior statin use (good 25 vs. poor 15, *p* = 0.471), systolic blood pressure on admission (good 162 vs. poor 165, *p* = 0.222), diastolic blood pressure on admission (good 97 vs. poor 95, *p* = 0.741) between the two groups (Table 1).

Five hundred and fifty-five (84.9) patients were given blood pressure (BP) lowering treatment (intravenous or oral) after admission. However, the intensity of BP-lowering was not recorded in the database.

### Correlations Between Non-HDLC Levels and 3-Month Functional Outcome

Prior to adjusting for any possible confounders, high non-HDLC levels (≥125.39 mg/dl) were associated with good 3-month functional outcomes (non-HDLC 125.39–154.89 mg/dl, OR 0.62, 95%CI, 0.40–0.96; non-HDLC ≥ 154.89 mg/dl, OR 0.49, 95%CI, 0.31–0.76; *p*-value for trend = 0.001). After adjusting for age, sex, history of ICH, NIHSS, and GCS scores on admission, glucose on admission, WBC on admission, platelets on admission and hematoma volume, statins use after admission and ICH score, high non-HDLC (≥154.89 mg/dl) remained as an indicator of good 90-day functional outcomes (multivariate-adjusted OR 0.50, 95%CI 0.27–0.92; *p*-value for trend = 0.043) (Table 2).

**Table 2:** Multivariate-adjusted OR and 95% CI for poor outcome (mRS ≥ 3) according to quartile of Non-HDLC.

	**Quartile of non-HDLC (mg/dl)**				
	**Q1 (<101.78)**	**Q2 (101.78–125.39)**	**Q3 (125.39–154.89)**	**Q4 (≥154.89)**	***p*-value for trend**
Unadjusted					0.001
OR(95%CI)	1.00 (Reference)	0.78 (0.51–1.21)	0.62 (0.40–0.96)	0.49 (0.31–0.76)	
*p*		0.272	0.031	0.002	
Adjusted^*^					0.043
OR (95%CI)	1.00 (Reference)	0.68 (0.38–1.23)	0.73 (0.39–1.35)	0.50 (0.27–0.92)	
*p*		0.197	0.312	0.027	

*CI, confident interval; non-HDLC, non-high-density lipoprotein cholesterol; OR, odd ratio*.

* *Adjusted for age, sex, History of ICH, NIHSS score on admission, GCS score on admission, Glucose on admission, WBC on admission, Platelets on admission, Hematoma volume, Statins use after admission, ICH score*.

Further analysis of the interaction effects of age and sex on the association between non-HDLC levels and the 90-day clinical outcome showed that there was a significant difference between males and females (*p* for interaction = 0.024). Female patients with high non-HDLC levels (≥154.89 mg/dl) were less likely to have poor 90-day clinical outcome (OR 0.13, 95%CI 0.03–0.50); however, we did not observe this tendency in male patients (OR 0.83, 95%CI 0.39–1.74). Age had no interaction effect on the association between non-HDLC levels and the 90-day clinical outcome (*p* interaction > 0.05), although some OR values were significant in subgroups (*p* < 0.05) (Table 3).

**Table 3:** Multivariate-adjusted^#^ OR and 95% CI for poor outcome (mRS ≥ 3) according to quartile of Non-HDLC, stratified by age, and gender.

	**Quartile of non-HDLC (mg/dl)**				
	**Q1 (<101.78)**	**Q2 (101.78–125.39)**	**Q3 (125.39–154.89)**	**Q4 (≥154.89)**	***p* interaction**
Age					0.406
<60 y	1	0.97 (0.42–2.32)	0.71 (0.30–1.72)	0.63 (0.26–1.50)	
≥60 y	1	0.51 (0.21–1.21)	0.68 (0.26–1.76)	0.36 (0.14–0.92)^*^	
Gender					0.024
Male	1	0.82 (0.37–1.69)	0.73 (0.34–1.58)	0.83 (0.39–1.74)	
Female	1	0.53 (0.15–1.87)	0.54 (0.15–1.95)	0.13 (0.03–0.50)^*^	

*CI, confident interval; non-HDLC, non-high-density lipoprotein cholesterol; OR, odd ratio*.

# Adjusted for age, sex, History of ICH, NIHSS score on admission, GCS score on admission, Glucose on admission, WBC on admission, Platelets on admission, Hematoma volume, Statins use after admission, ICH score.

* *p < 0.05*.

We also performed multivariate analysis according to every 10 mg/dl increase in non-HDLC. We found that serum non-HDLC level is an independent protective factor against poor functional outcomes at 90 days from onset after adjusting for age, sex, history of ICH, NIHSS and GCS scores on admission, glucose on admission, WBC on admission, platelets on admission, hematoma volume, statins use after admission, and ICH score. The poor prognosis risk at 90 days was reduced by 2.8% for every 10 mg/dl increase in non-HDLC (Table 4).

**Table 4:** Multivariate-adjusted^#^ OR and 95% CI for poor outcome (mRS ≥ 3) according to every 10 mg/dl of non-HDLC.

	**OR (95%CI)**	***p***
Gender	1.234 (0.776–1.963)	0.374
History of ICH	1.649 (0.370–7.349)	0.512
Admission GCS score^*^	1.223 (1.062–1.408)	0.005^*^
Admission NIHSS score^*^	1.255 (1.192–1.322)	<0.001^*^
Admission WBC^*^	1.101 (1.029–1.178)	0.005^*^
Admission platelets	0.997 (0.993–1.001)	0.110
Admission glucose	1.050 (0.981–1.124)	0.158
non-HDLC (10mg/dl)^*^	0.937 (0.884–0.993)	0.028^*^
Hematoma volume	0.998 (0.983–1.014)	0.808
ICH score^*^	1.375 (1.004–1.883)	0.047^*^
Age (<60 y vs. ≥60 y)^*^	2.918 (1.883–4.522)	<0.001^*^

*CI, confident interval; GCS, Glasgow Coma Scale; ICH, intracerebral hemorrhage; mRS, modified Rankin Scale; NIHSS, National Institutes of Health Stroke Scale; non-HDLC, non-high-density lipoprotein cholesterol; OR, odd ratio; WBC, white blood cells*.

# *Adjusted for age, sex, History of ICH, NIHSS score on admission, GCS score on admission, Glucose on admission, WBC on admission, Platelets on admission, Hematoma volume, Statins use after admission, ICH score*.

* *p < 0.05*.

## Discussion

In this prospective study, we observed a decreasing tendency in the proportion of poor prognoses at 90 days after ICH as non-HDLC levels increased. The results suggested that elevated non-HDLC level is an independent indicator for the good functional outcomes after ICH, especially when non-HDL level reached 154.8 mg/dl and above.

The original study of the influence of serum non-HDLC levels was in the field of cardiovascular disease (15). Though several studies (16–18) has proposed that serum non-HDL-C levels are closely associated with cerebrovascular diseases, studies about the association between serum non-HDLC level and ICH are limited. Previous studies showed there is a correlation between serum cholesterol levels and the occurrence and development of ICH (19, 20). For example, serum TC levels are inversely associated with the death risk from ICH (21) and lower levels of LDL are associated with higher ICH risk (22, 23). Chang et al. found that low LDL levels at admission were independent risk factors for hematoma enlargement and in-hospital deaths in ICH patients (19). However, these studies did not mention whether serum non-HDLC level has protective effect in ICH patients.

Conclusions from studies explored the impact of serum non-HDL level on ICH occurrence remains controversial. In the Kailuan Study (95,916 Chinese participants), researchers found higher serum non-HDLC levels were associated with increased risks for total stroke and ischemic stroke, but not for intracerebral and subarachnoid hemorrhage (9). In a Japanese study (24), they showed lower non-HDLC levels were associated with an increased risk of ICH, particularly lobar ICH.

In our study, we found non-HDLC levels are associated with the prognosis of ICH for the first time and confirmed the correlation between serum cholesterol levels and ICH.

In addition, we found that the relationship between non-HDLC levels and the prognosis of patients with ICH may differ between males and females. In our study, we observed an interaction effect from sex on the association between non-HDLC levels and 90-day clinical outcomes. Our results suggest non-HDLC levels are more likely to affect females with ICH. Stroke has a greater effect on females than males because females have more stroke events and are less likely to recover (25). A study of the relationship between female lipid levels and ICH suggested LDL levels <70 mg/dl and low TC levels were associated with increased risk of hemorrhagic stroke (20). Our results are in agreement with previous studies that gender differences appear in the association between hypertension and stroke risk and that hypertension is more likely to cause ischemic stroke in women (26, 26). Sheth et al. found after endovascular therapy for acute ischemic stroke, female patients had experienced better functional outcomes and more years of optimal life compared with male patients (27). These results suggest differences in the effects of stroke risk factors on populations of different sexes. Our results are consistent with previous conclusions and further studies are needed to confirm the sex difference in the effect of non-HDLC levels on ICH outcomes. The sex differences in the effect of non-HDLC level on ICH prognosis might be caused by following reasons: Firstly, since the majority of patients recruited were over 48 years old, and could be influenced by the estrogen level reduction. Estrogen is a protective factor against vascular atherosclerosis (28–30) and females have a nearly decade-long delay to their first myocardial infarction compared to males (31). Studies found atherosclerosis were closely related to cerebral microbleeds, when the atherosclerosis degree increased, the averaged cerebral microbleeds number increased (32, 33). Elder women (losing the of estrogen protection) might have higher degree of atherosclerosis and be more likely to have multiple cerebral microbleeds. Moreover, a study confirmed higher LDL levels were associated with lower mortality in patients with ICH (19), and the underlying mechanism could be the association between high levels of LDL and fewer cerebral microbleeds (34). Non-HDLC may function similarly to LDL. Although there is no sufficient evidence to show the relationship between cerebral microbleeds and ICH prognosis, studies have shown that cerebral microbleeds are closely related to poor outcome of acute ischemic stroke patients treated with intravenous thrombolysis (35). We speculated high levels of non-HDLC would reduce the severity of cerebral microbleeds in females and ameliorate poor functional outcome. The specific mechanisms still need to be confirmed. These mechanisms may explain why the association between non-HDLC levels and the prognosis of ICH was more significant in females in our findings. Secondly, we consider that differences in lipid metabolism vary with sex due to genetic differences (36–39).

However, the potential limitations also merit consideration. First, all participants were from the same area, which might limit the application of the findings to a population with broader geographic and ethnic diversity. Second, we did not record the information about the intensity of BP-lowering, so there was an absence of adjustment for the intensity of BP-lowering in multivariate analysis. This need to be further improved in the future studies.

The present study assesses the relationship between non-HDLC levels and 90-day clinical outcome. Our findings suggest that monitoring non-HDLC levels in patients with ICH could be helpful in predicting their prognosis. Further studies are needed to explore the mechanism and clinical importance of non-HDLC in ICH patients, with possible implications for cerebrovascular disease prevention.

## Conclusion

In summary, ICH patients with higher non-HDLC levels had a decreased prevalence of poor functional outcome at 90 days, and a high non-HDLC level is an independent indicator of good functional outcome at 90 days from onset, especially in female.

## Data Availability Statement

The datasets analyzed in this manuscript are not publicly available. Requests to access the datasets should be directed to zxq@vip.163.com.

## Ethics Statement

The study protocol was approved by the Institutional Review Board (IRB) of the Beijing Tiantan Hospital affiliated to Capital Medical University. The patients/participants provided their written informed consent to participate in this study.

## Author Contributions

HF, XW, and XZ designed the study and drafted the manuscript. HF and WW collected the clinical data and managed the database. All authors approved for the manuscript submitted.

## Conflict of Interest

The authors declare that the research was conducted in the absence of any commercial or financial relationships that could be construed as a potential conflict of interest.
